# Bibliometric analysis: a study of the chronic atrophic gastritis in gastric cancer (2015–2024)

**DOI:** 10.3389/fonc.2025.1576823

**Published:** 2025-06-09

**Authors:** Xu-Li Liu, Li-Li Li, Ji-Lan Wan, Li-Qian Chen, Mai-Qing Yang

**Affiliations:** ^1^ Department of Special Inspection, Changyi People’s Hospital, Changyi, Shandong, China; ^2^ Department of Pathology, Weifang People’s Hospital (First Affiliated Hospital of Shandong Second Medical University), Weifang, Shandong, China

**Keywords:** gastric cancer, chronic atrophic gastritis, bibliometric analysis, VOSviewer, CiteSpace

## Abstract

**Objective:**

Gastric cancer (GC) is common worldwide and the fifth leading cause of cancer-related deaths. Chronic atrophic gastritis (CAG) is a precancerous stomach lesion closely associated with GC. This study aimed to conduct a comprehensive analysis of global CAG research in GC and provide a knowledge framework from a holistic and systematic perspective based on bibliometric analysis.

**Methods:**

Studies focusing on CAG in GC were performed using the Web of Science Core Collection database. The annual output, cooperation, hotspots, research status, and development trends in this field were analyzed using bibliometric software (VOSviewer and CiteSpace).

**Results:**

A total of 1,065 articles published between 2015 and 2024 were selected. Recently, the number of publications and citations has increased. Cooperation network analysis indicated that China holds the foremost position in the research on CAG in GC, with the highest volume of publications and citations, thus exerting the greatest influence. China Medical University had the highest research output. Additionally, *World Journal of Gastroenterology* has reported the highest degree of productivity in this field. Yuan Yuan was the top contributor and the most frequently co-cited author. Cluster analysis of the authors’ keywords identified four key areas: *Helicobacter pylori*, atrophic gastritis, dysplasia, biomarkers and artificial intelligence, which have attracted increasing attention from researchers.

**Discussion:**

This bibliometric analysis provides a data-based and objective introduction to CAG in GC and offers readers a valuable reference for future research.

**Conclusions:**

Our study systematically summarizes the results of CGA in GC research (2015–2024) and describes and predicts research hotspots and trends on a global scale. Mechanisms and therapies of CAG in GC remain key future research topics.

## 1 Introduction

Gastric cancer (GC) is the fifth most frequently diagnosed cancer globally and the fifth leading cause of cancer-related deaths (1). Most (approximately 90%) GCs are adenocarcinomas arising from the glands of the most superficial layer of the stomach (2). Risk factors for this condition include *Helicobacter pylori* (HP) infection, age, high salt intake, and diets low in fruits and vegetables (3). Even with perioperative and adjuvant chemotherapies/chemoradiation therapies, the 5-year disease survival rates sharply drop for patients beyond Stage II, ranging from 61–63% for Stage IIIa to 30% – 35% for Stage IIIc (4). The complex and treacherous nature of GC often leads to late diagnosis and poor prognosis in advanced stages, making strategies for prevention and early detection of great significance.

Chronic atrophic gastritis (CAG), on the other hand, is recognized as a crucial precursor lesion in the gastric carcinogenesis pathway (5). The condition is characterized by gastric mucosal atrophy. Two types of CAG have been reported: a predominantly gastric body type in patients with HP infection and an autoimmune type limited to the gastric body and fundus (5, 6). However, the autoimmune type is rare. HP infection is highly prevalent and exhibits significant geographical variation. It is less commonly seen in high-income countries. Owing to the substantial number of patients who eventually progress to GC, HP infection is recognized as a carcinogen (7–10).

Recently, several studies have been conducted on CAG in GC (11, 12), with an increasing number of researchers focusing on this topic. However, the vast expanse of publications renders it highly difficult for researchers to remain current with the cutting-edge developments and distinguish the most consequential contributions. A systematic and all - encompassing literature review is of great significance in enabling a thorough comprehension of current research and guiding future research directions. Bibliometrics is the analysis of publications using statistics to describe or display relationships among published works (13, 14). The Web of Science Core Collection (WOSCC) is a widely recognized, comprehensive academic literature database that includes references cited in papers and compiles unique citation indices based on cited authors, sources, and publication years (15). Many studies have employed this database for bibliometric analyses. To the best of our knowledge, no bibliometric analyses focusing on CAG in GC have been published. In this study, we aimed to use a quantitative approach to analyze CAG in GC treatment, identify the key contributors and current status of research in the field, as well as propose future research trends.

## 2 Materials and methods

### 2.1 Data collection

**Figure 1:**
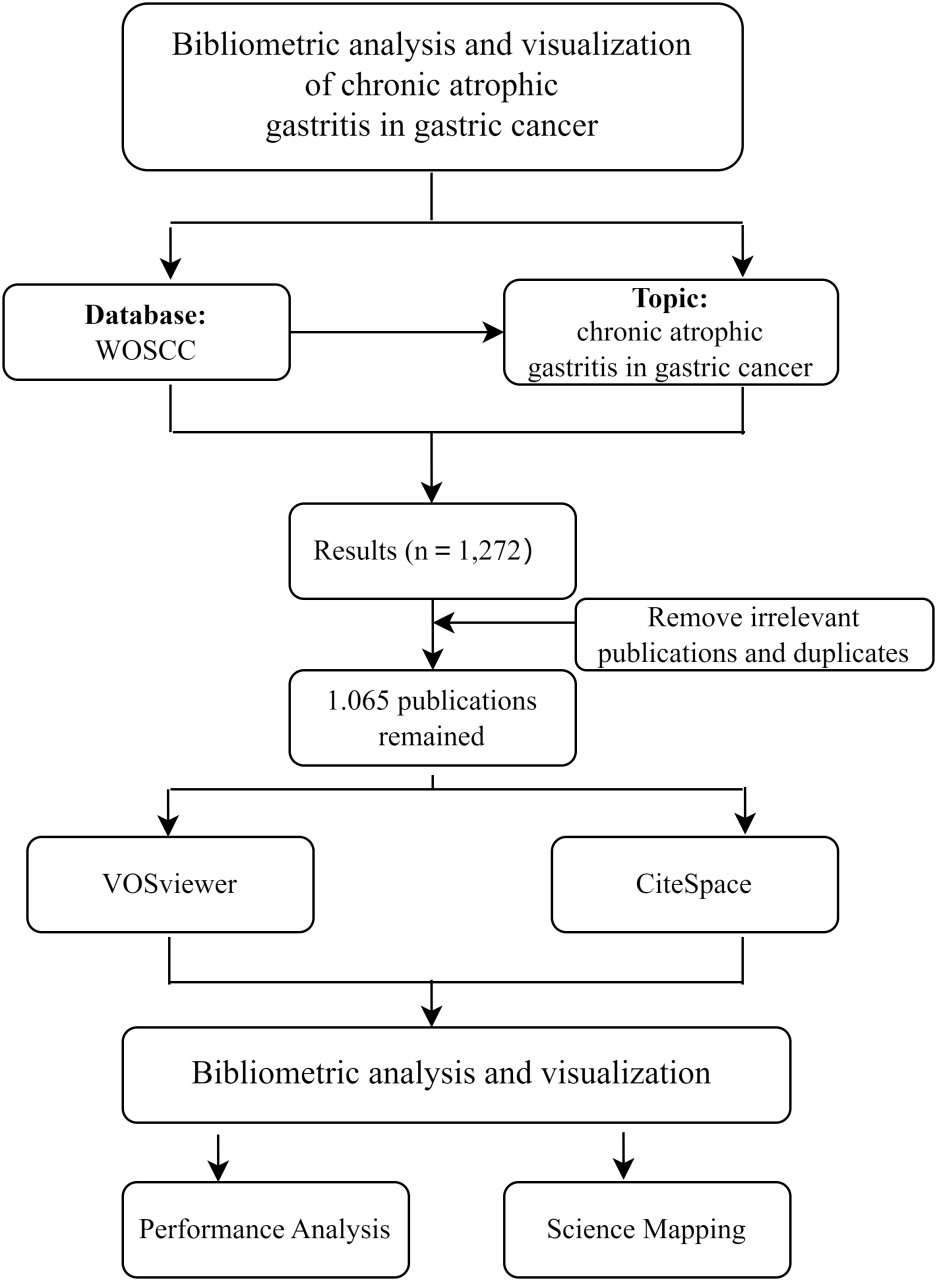
Study flow chart.

We performed a literature search of the WOSCC (https://www.webofscience.com/wos/woscc/basic-search). The search was conducted on January 26, 2025. The publication period for this study is between 2015 and 2024. The search terms were presented as follows: Topic= “atrophic gastritis” AND Topic = “gastric cancer” OR “gastric carcinoma” OR “gastric adenocarcinoma” OR “gastric malignancy” OR “gastric neoplasm” OR “stomach cancer” OR “stomach carcinoma” OR “stomach adenocarcinoma” OR “stomach malignancy” OR “stomach neoplasm.” After the preliminary search, publications were screened based on the following inclusion criteria: (1) Articles were published between January 1, 2015, and December 31, 2024. (2) English-language articles and reviews were included. All documents related to CAG and GC were exported in “full records and references” TXT format. Inclusion criteria focused on research topics that were directly related to the CAG in GC. Exclusion criteria were specified, and the retrieved documents were deduplicated. Two people conducted a preliminary screening of the retrieved studies according to pre-established inclusion and exclusion criteria. If there is a disagreement between the two people, a consensus will be reached through discussion. VOSviewer and CiteSpace were retrieved and imported for the bibliometric analysis (16, 17). The search tactics are illustrated in **Figure 1**.

### 2.2 Data analysis and visualization

The software tools utilized for the bibliometric analysis were VOSviewer and CiteSpace. VOSviewer (version 1.6.20, Leiden University, The Netherlands) was used to create visual graphs and analyze the most cooperative countries, institutions, and authors, as well as the most co-cited journals and co-occurring keywords. CiteSpace software (version 6.3. R1; Drexel University, PA, USA) was employed to generate timeline graphs and bursts of keyword terms. Keyword emergence refers to the sudden and frequent use of keywords within a certain period to reflect hot topics and future trends in a research field (18, 19). The different nodes represent various elements. Each dot on the visual graph represents a country, institution, author, or journal, and these dots are clustered into different groups based on cooperation. Moreover, the dot sizes reflect the total frequency of each element. The connecting lines between nodes represent co-occurrence, cooperation, or co-citation (20, 21). In this study, variables were expressed as numbers and percentages. No comparisons were made; therefore, no P values were established.

## 3 Results

### 3.1 Analysis of publication quantity and trends

A total of 1,272 related research documents and 1,065 pieces of literature related to the involvement of CAG in GC were included in the analysis after selection. Among these, 829 were original research articles, whereas 236 were reviews (**Table 1**). **Table 1** presents the annual distribution of articles. Publication volume steadily increased (2015–2019), then surged (2020–2024), reflecting a heightened focus on this area.

**Table 1:** Annual distribution of the number of papers (2015-2024).

Year	Papers	% of 1,065
2024	122	11.46%
2023	126	11.83%
2022	138	12.96%
2021	111	10.42%
2020	112	10.52%
2019	98	9.20%
2018	96	9.01%
2017	104	9.77%
2016	90	8.45%
2015	68	6.38%

### 3.2 Distribution of countries and institutions

#### 3.2.1 Contributions of countries

**Figure 2:**
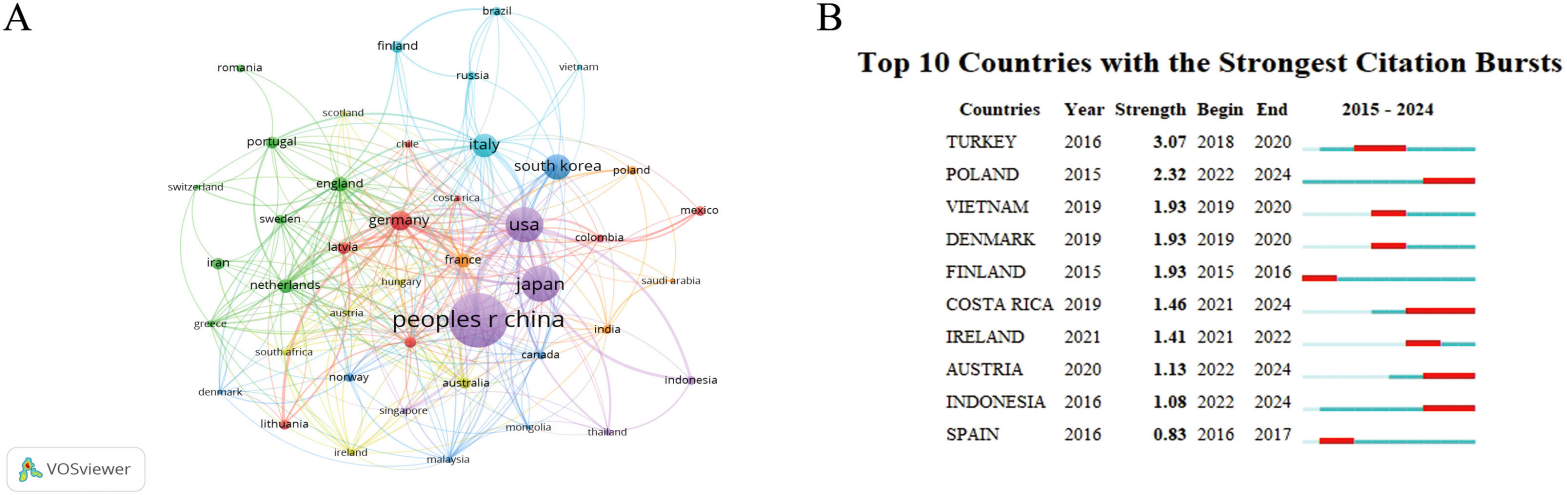
Visualization of countries. **(A)** Network visualization map of co-citations of countries. **(B)** Top 10 countries with the strongest citation bursts.

We employed the VOSviewer software to analyze the data and generate national visualization maps (**Figure 2A**). The results demonstrated that the field of CAG in GC attracted the attention of 81 countries, the top five countries for the number of papers published in this field were China (398, 37.37%), Japan (173, 16.24%), the United States (USA) (166, 15.59%), South Korea (91, 8.54%), Italy (76, 7.14%); the top five centrally ranked countries were China, USA, Japan, Italy and South Korea (**Table 2**; **Figure 2**). These results indicate China’s leading position in this field. **Figure 2B** lists the top 10 cited countries ranked by the duration of their citation bursts, highlighting their significant impact.

**Table 2:** The top five countries with the most reviews and total citations.

Number	Countries	Document	Citations
1	China	398	7,240
2	Japan	173	5,115
3	The United States	166	6,922
4	South Korea	91	2,072
5	Italy	76	2,716

#### 3.2.2 Contributions of institutions

**Figure 3:**
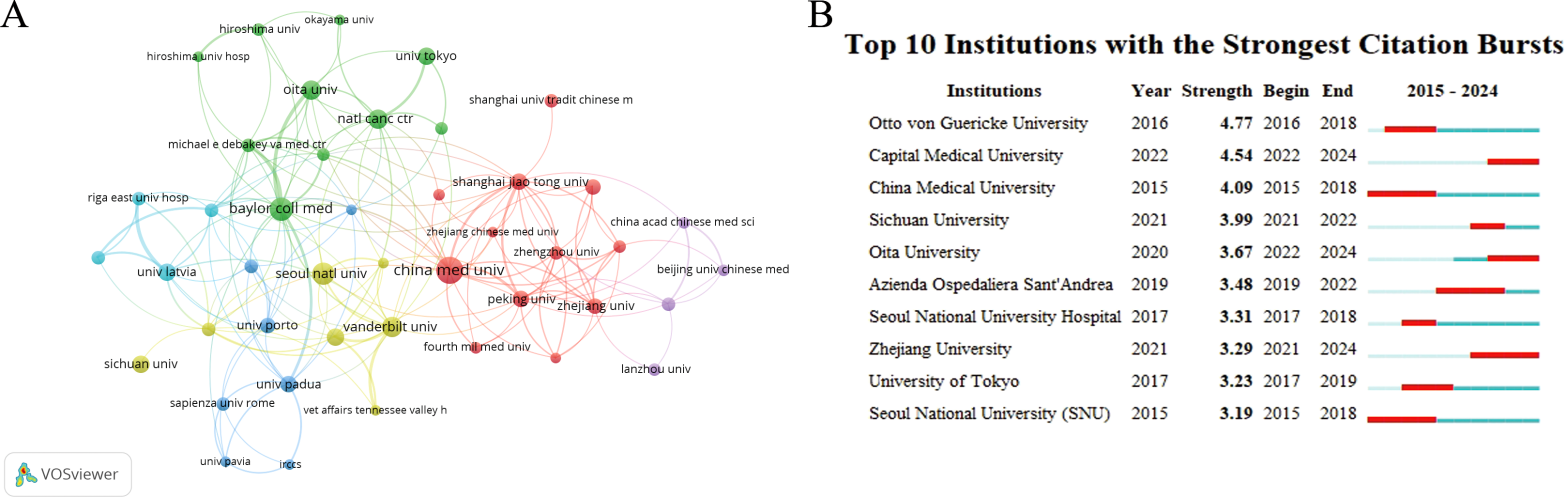
Visualization of institutions. **(A)** Network visualization map of co-citations of institutions. **(B)** Top 10 institutions with the strongest citation bursts.

The field of CAG in GC has attracted the attention of 1,741 academic institutions. **Figure 3A**, which was generated using VOSviewer, demonstrates an institutional visualization map. China Medical University was highlighted as the foremost contributor with 48 publications; other prominent institutions included the Baylor College of Medicine, Seoul National University, Vanderbilt University, and Oita University (**Table 3**). Institutional analysis revealed that institutions with significant collaborations were mainly from China (**Figure 3A**). **Figure 3B** ranks the top 10 cited institutions by the duration of their citation bursts, highlighting the significant impact of these institutions.

**Table 3:** The top 10 institutions with the most reviews and total citations.

Number	Institutions	Document	Citations
1	China medical university	48	1,219
2	Baylor college of medicine	35	2,686
3	Seoul national university	32	1,115
4	Vanderbilt university	28	1,749
5	Oita university	24	598
6	National cancer center	24	342
7	National college of Ireland	20	555
8	University of Tokyo	20	520
9	University of Latvia	19	890
10	Sichuan university	19	398

### 3.3 Analysis of authors

**Figure 4:**
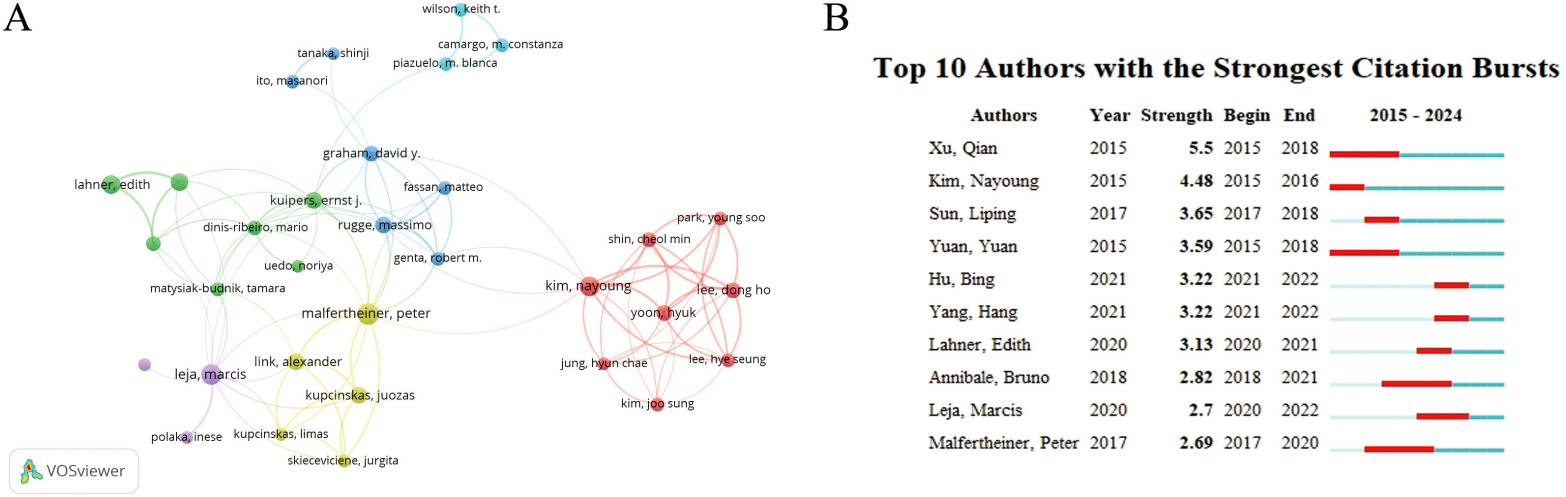
Visualization of authors. **(A)** Network visualization map of co-citations of authors. **(B)** Top 10 authors with the strongest citation bursts.

Examining the seminal works of impactful authors in a given field provides a way to comprehend classical theory. In total, 5,911 authors contributed to 1,065 publications, yielding a co-authorship index of 5.55. **Table 4** lists authors who published 14 or more research articles with more than 300 citations. The results demonstrated that Yuan Yuan and Xu Qian from China Medical University in China, Malfertheiner Peter from LMU, Munchen in Germany had the most prolific outputs and citations. **Figure 4** illustrates a dense author co-citation network, highlighting the top 10 cited authors’ impact.

**Table 4:** The top authors with the most reviews and total citations.

Number	Authors	Document	Citations
1	Yuan, yuan	37	718
2	Xu, qian	26	507
3	Malfertheiner, peter.	21	1,742
4	Leja, marcia	19	903
5	Kim, nayoung	17	688
6	Lahner, edith	15	460
7	Sun, liping	15	367
8	Annibale, bruno	14	1,010

### 3.4 Analysis of journals

**Figure 5:**
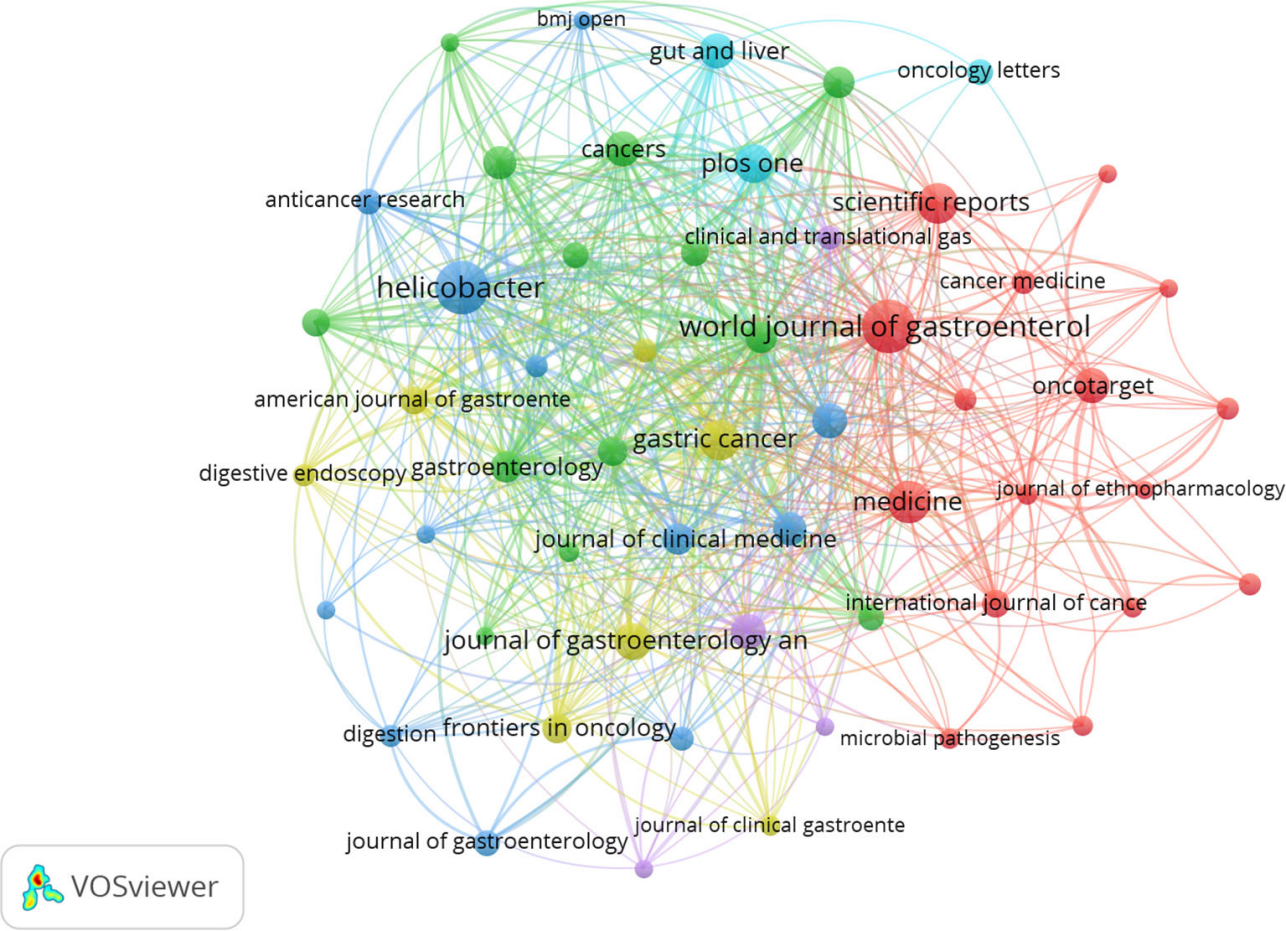
Visualization of journals. Network visualization map of co-citations of journals.

A total of 354 journals have published articles or reviews in this field. **Table 5** lists the top 15 journals by publication count. *World Journal of Gastroenterology* was the most prolific journal, with 39 articles, followed by *Helicobacter* (38), *Medicine* (25), *Scientific Reports* (23), and *Gastric Cancer* (22). Additionally, *Gut* had the highest citation rate (2,984 citations). **Figure 5** displays a journal co-citation network map. These results indicate the high quality of the articles published in these journals and their high scientific value and impact. This guides researchers in selecting journals for manuscript submission. In addition, researchers can follow these journals to update their relevant research.

**Table 5:** The top 15 journals with the most reviews and total citations.

Number	Journals	Document	Citations
1	World journal of gastroenterology	39	1,059
2	Helicobacter	38	1,152
3	Medicine	25	175
4	Scientific reports	23	532
5	Gastric cancer	22	565
6	Plos one	20	414
7	Journal of gastroenterology and hepatology	18	319
8	Scandinavian journal of gastroenterology	17	482
9	Gut and liver	16	385
10	Oncotarget	16	359
11	International of journal of molecular sciences	16	341
12	Cancers	16	209
13	Digestive diseases and sciences	15	205
14	BMC gastroenterology	15	143
15	Gut	14	2,984

### 3.5 Key topics of research hotspots

Keywords represent the core of a scientific paper. Thus, by analyzing keywords, we can track knowledge evolution, hotspots, and future research directions. This study explores research hotspots widely studied over a specific period.

#### 3.5.1 Analysis of clusters and co-occurrence of keywords

**Figure 6:**
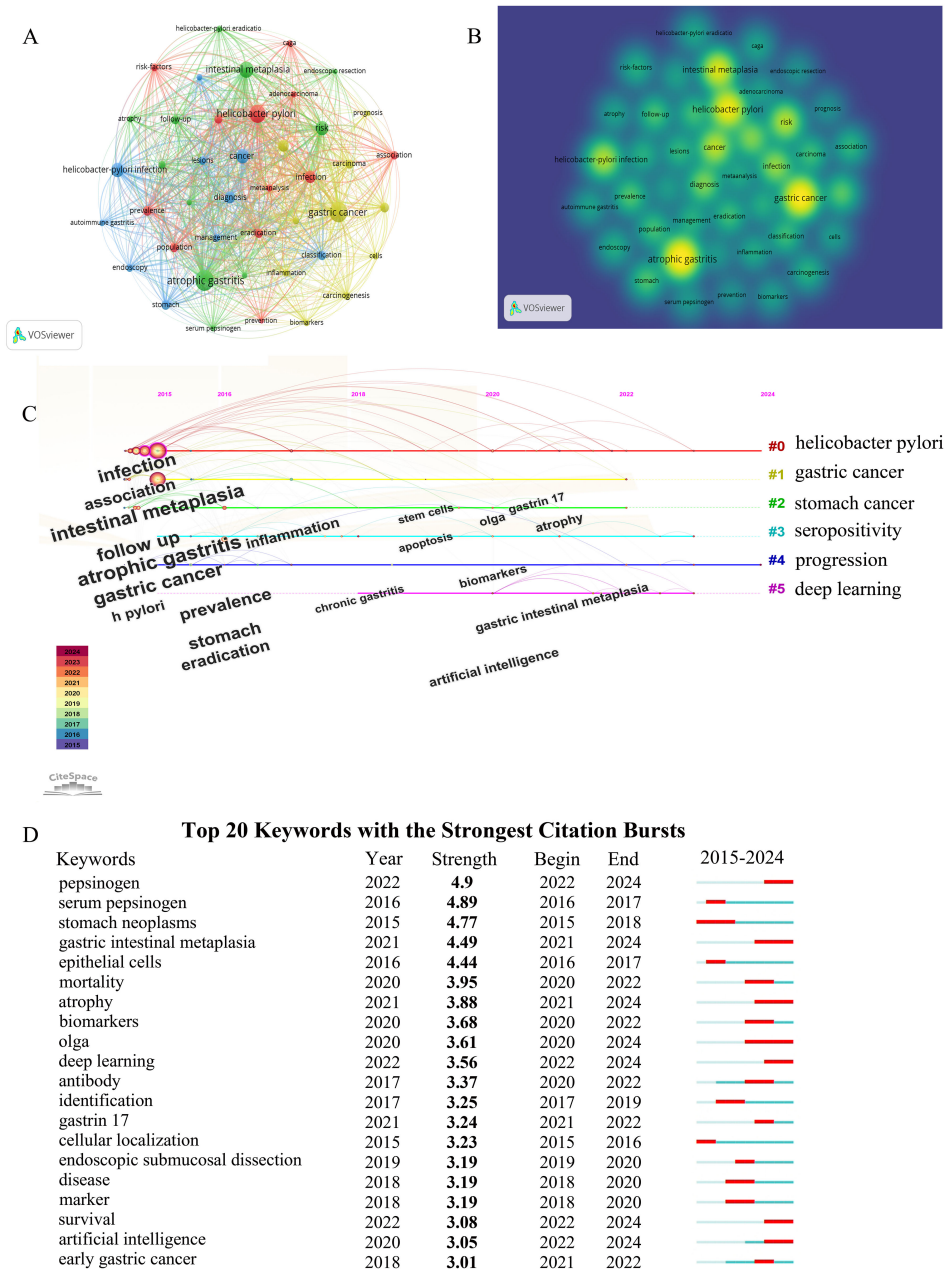
Visualization of keywords. **(A)** Network visualization map of co-citations of keywords. **(B)** Analysis results of hotspot and topic migration in the field of tumor microenvironment in gastric cancer. **(C)** Keyword clustering timeline graph. **(D)** Top 20 keywords with the strongest citation bursts.

VOSviewer was utilized to draw a keyword co-occurrence network visualization of the 1,065 articles. A total of 3,625 keywords were included in this study. Based on the link strength of keyword co-occurrence, we selected 46 keywords with a frequency of occurrence ≥ 30 times for visualization and divided the network into four clusters. The concept of different research directions on a topic is proposed through a statistical analysis of keywords in various parts of the paper. Cluster analysis of keywords provided insights into the knowledge structure of this field. Clusters 1 (red) and 2 (green) were the largest, with 12 and 11 terms, respectively. The main themes in cluster 1 (red) were adenocarcinoma, association, epidemiology, eradication, and HP infection. Cluster 2 (green) was primarily associated with atrophic gastritis, atrophy, endoscopic resection, follow-up, and intestinal metaplasia, meanwhile, cluster 3 (blue) focused on autoimmune gastritis, cancer, classification, diagnosis, and dysplasia. Cluster 4 (yellow) was associated with biomarkers, carcinogenesis, cells, inflammation, prognosis and artificial intelligence (**Figures 6A, B**).

#### 3.5.2 Burst detection and overlay visualization of keywords

The keywords from 1,065 articles were analyzed to identify burst words. Burst word analysis highlights hot research topics within a specific period. **Figures 6C, D** demonstrate the strongest keyword bursts in CAG for GC research between 2015 and 2024. In the figure, the year represents the earliest appearance of a keyword. Begin and End indicate the start and end times of the burst, respectively. The red bar denotes the times when keywords appear frequently, whereas the blue bar demonstrates the times when keywords occur infrequently (22). “Pepsinogen” was the most robust burst keyword (strength 4.9, from 2022 to 2024), followed by “serum pepsinogen” (strength 4.89, from 2016 to 2017), “stomach neoplasms” (strength 4.77, from 2015 to 2018), “gastric intestinal metaplasia” (strength 4.49, from 2021 to 2024), and “epithelial cells” (strength 4.44, from 2016 to 2017), revealing that these keywords represented the popular research topics in recent years and even in the near future.

## 4 Discussion

### 4.1 Summary of findings

This analysis software offers a valuable reference for researchers in the field of GC. All documents on a particular subject within a limited period that contain a variety of effective information were analyzed and quantified to summarize the effective information. This reveals whether a scientific topic was a hotspot for a certain period and can be used to predict its future trends.

In this study, 1,065 articles published between 2015 and 2024 were included. The results indicate that the annual number of publications has increased. Between 2015 and 2019, the number of articles published was moderate with an average annual publication of 91.2 papers. Furthermore, between 2020 and 2024, the number of publications increased, with an average of 121.8 papers. Many researchers have focused on this field and journals are increasingly paying attention to it. In this research field, China and Japan have the deepest academic accumulation and the greatest influence. China demonstrated the highest level of international collaboration and had the highest publication count. Our results indicated that China played a leading role, with China Medical University being the most productive institution worldwide. Three of the top eight authors in this field were Chinese: Yuan Yuan, Xu Qian, and Sun Liping. The findings underscore the remarkable contributions and latent scientific innovations made by Chinese researchers in the field of CAG in GC. Yuan Yuan from China Medical University published the largest number of articles and had the highest centrality, indicating that the academic outcomes of his collaborations were prominent and had considerable influence in the field. Authors from various countries should strengthen their cooperation to promote the development of this field.

The top three countries contributed two-thirds of the total publications. Approximately 97% of the affiliations had no more than five articles. This indicates that most affiliations in this field have not made in-depth investments and only a few affiliations have performed continuous research. Therefore, seeking extensive collaborations among institutions is important. Many journals have been concerned with this field. Additionally, *World Journal of Gastroenterology* was the most productive. Current research on CAG in GC is mainly published in oncology-, biology-, genetics-, and medicine-related journals. This highlights the field’s broad scope, and its development from basic to clinical research.

### 4.2 Research hotspots and frontier exploration in CAG of GC

The development of keywords over time reflects the evolution of cutting-edge knowledge. This study will play an important role in guiding future research by detecting burst keywords to identify research fronts. Burst keywords indicate keywords that have been widely cited over time. From the results, “pepsinogen” was the most robust burst keyword (strength 4.9, from 2022 to 2024), followed by “serum pepsinogen” (strength 4.89, from 2016 to 2017), “stomach neoplasms” (strength 4.77, from 2015 to 2018), “gastric intestinal metaplasia” (strength 4.49, from 2021 to 2024), and “epithelial cells” (strength 4.44, from 2016 to 2017). The research structure in this field can be summarized using a keyword co-occurrence network. The first part explored the relationship between CAG and GC, followed by mechanism research on topics, such as “*helicobacter pylori*,” “inflammation,” and “biomarker.” Practical application research has also been conducted on deep learning and artificial intelligence.

### 4.3 Main focus of research of CAG in GC

The analysis demonstrated that the principal research topics within the context of CAG in GC extend across diverse domains. A number of publications delved into the underlying mechanisms that link CAG to the development of GC. These studies encompassed investigations into genetic mutations, abnormal RNA, as well as microbe-metabolic interactions (23–27). Another crucial area pertains to the research on traditional Chinese medicine treatments (12, 28), which expounded on the potential mechanisms, such as pathway blocking and molecular dynamics simulation (24). Considerable attention has been devoted to the identification of dependable biomarkers for the early detection of GC. Serum gastrin-17, pepsinogen, and Trefoil Factor 3 have emerged as potential predictors during the asymptomatic stage of GC (23, 29, 30). Additionally, artificial intelligence has been harnessed to achieve early and precise detection of CAG (31).

Comprehending the research patterns across various regions constitutes a significant focal point as well. China, as one of the regions with an exceptionally high incidence rate of GC, has furnished a considerable number of studies. Nevertheless, it is of great importance to acknowledge that such profusion might predominantly originate from the local epidemiological requirements and not necessarily reflect the global panorama in a homogeneous manner.

East Asia, eastern Europe, and Latin America have the highest incidences in GC, while North America and most of western Europe have relatively low incidences (32). Differences also emerge in research fields. In high-incidence areas such as certain parts of East, where CAG is firmly established as a precursor closely associated with the intestinal subtype of GC, research endeavors have been intensively focused on deciphering the sequential progression from CAG to the development of the intestinal subtype (33). The prevalence of HP infection varies markedly across different geographical regions, and HP infection notably augments the cancer risk, especially for intestinal-type distal carcinoma (9). This has spurred investigations into the role of HP infection, dietary factors, and the intricate interplay of inflammatory mediators in facilitating epithelial metaplasia and subsequent tumorigenesis (9, 34). In China specifically, there has been a substantial body of research dedicated to the exploration of traditional Chinese medicine treatments (12, 28). Conversely, in Western populations, where the diffuse subtype of GC is becoming increasingly prominent, research trends are gravitating towards distinct aspects, with studies concentrating on specific gene mutations and the immune microenvironment (35, 36). This comparative methodology enriches our understanding of the intricate mosaic of GC research across diverse geographical regions and lays the groundwork for more collaborative and impactful studies in the future.

### 4.4 Future development

Numerous studies have been conducted on CAG in GC to build a solid foundation for this field; however, it is still at a superficial level. Exploring the origin of gastric metaplasia is a popular topic in the field of CAG.

### 4.5 Limitations

This study provided a comprehensive bibliometric analysis of CAG in GC, which has several limitations that may impact the results. The data used in this study were obtained solely from the WOSCC database, which may have excluded valuable information. Only articles and reviews were included; other social publications such as editorials and books were not considered. Although our search strategy was designed to be comprehensive, some relevant keywords may have been overlooked, which may have affected our results.

## 5 Conclusions

Through a systematic literature search, we identified that this is the first study to analyze the literature on CGA in GC using a bibliometric approach. Compared to traditional reviews, the study not only contains information on countries, institutions, authors, and journals but also specifically illustrates the internal relationship between keywords in different clustering groups and their influence on CGA in GC.

## Data Availability

The original contributions presented in the study are included in the article/supplementary material. Further inquiries can be directed to the corresponding author.
